# Biosimilar Nivolumab-Based Combination Therapy in Advanced Oral Cavity Squamous Cell Carcinoma: A Real-World Case Series of Five Patients

**DOI:** 10.7759/cureus.112146

**Published:** 2026-07-06

**Authors:** Santhosh Meedimale, Soumya S Panda, Vipulkumar Thummar, Harsh Bhavsar

**Affiliations:** 1 Department of Medical Oncology, Omega Hospitals, Karimnagar, IND; 2 Department of Medical Oncology, Institute of Medical Sciences (IMS) and SUM Hospital, Siksha 'O' Anusandhan (SOA) Deemed to be University, Bhubaneswar, IND; 3 Department of Medical Affairs, Zydus Lifesciences Limited, Ahmedabad, IND

**Keywords:** head and neck neoplasms, immunotherapy, neoadjuvant therapy, nivolumab, squamous cell carcinoma

## Abstract

Squamous cell carcinomas of the head and neck (SCCHN) belongs to a biologically aggressive group of malignancies. The salvage options, after failure of the platinum-based therapy and immune checkpoint inhibitors (ICIs), are usually very limited. The regimen comprising oral methotrexate, erlotinib, and celecoxib (OMCT) has been explored as a well-tolerated, low-dose management option.

We present through the case series the clinical outcomes of patients who had recurrent, refractory, or locally advanced SCCHN treated with OMCT in combination with low-dose nivolumab for translatable evidence.

## Introduction

Squamous cell carcinomas of the head and neck (SCCHN) are globally estimated to account for 930,000 new cases and 467,000 deaths up to 2020 in the existing literature [1]. In India, these cancers are strongly linked to tobacco and betel nut use, particularly the oral cavity tumors (buccal mucosa and hard palate carcinomas), leading to a disproportionately high burden [2]. Locoregionally advanced and recurrent/metastatic (R/M) SCCHN carry a poor prognosis [3]. Standard first-line options include platinum-based doublets (cisplatin or carboplatin + 5-fluorouracil (5-FU)) with cetuximab (EXTREME regimen) [4] and immune checkpoint inhibitors (ICIs; pembrolizumab ± chemotherapy, KEYNOTE-048) [5]. Despite these advances, median overall survival in R/M disease remains 10-13 months, with few effective salvage strategies.

Nivolumab (anti-PD-1) has demonstrated OS benefit over single-agent chemotherapy in platinum-refractory SCCHN (CheckMate 141) [6], becoming a pivotal second-line standard. However, high-dose immunotherapy is costly, often inaccessible in resource-limited settings, and not universally feasible in patients with poor performance status (PS) or significant comorbidities [7, 8].

Metronomic chemotherapy, the administration of cytotoxic agents at low, continuous doses, targets the tumor microenvironment, exerts anti-angiogenic effects, and may potentiate ICI activity by modulating regulatory T-cells [9,10]. The oral methotrexate + erlotinib + celecoxib (OMCT) regimen exploits multiple non-overlapping mechanisms: antifolate cytotoxicity (methotrexate), EGFR inhibition (erlotinib) [11], and COX-2-mediated anti-inflammatory/pro-apoptotic effects (celecoxib) [12]. A low-dose nivolumab study, led by Dr. Vijay Patil and colleagues at Tata Memorial Hospital, adding ultra-low-dose nivolumab (20 mg) to metronomic chemotherapy safely doubles one-year survival in advanced head and neck cancer while cutting immunotherapy costs by roughly 90% [13]. Herein, we describe four consecutive patients with SCCHN treated with oral methotrexate (OMCT) 9 mg/m² once a week, celecoxib 200 mg twice daily, and erlotinib 150 mg once daily combined with low (40 mg every three weeks (q3w)) and standard dose (3 mg/kg every two weeks (q2w)) biosimilar nivolumab (Tishtha, Zydus Lifesciences Limited, Ahmedabad, India) and one case of low dose (40 mg q3w) biosimilar nivolumab (Tishtha) with neoadjuvant docetaxel, cisplatin, and fluorouracil (DCF) chemotherapy. These patients had no prior exposure to immunotherapy, and data were collected at nearly three months of follow-up post initiation. This is a retrospective case series, encompassing a spectrum of disease contexts: heavily pre-treated R/M disease, poor PS, patients ineligible for conventional chemotherapy, and neoadjuvant conversion therapy.

## Case presentation

Case 1: locally advanced carcinoma of the buccal mucosa 

**Figure 1:**
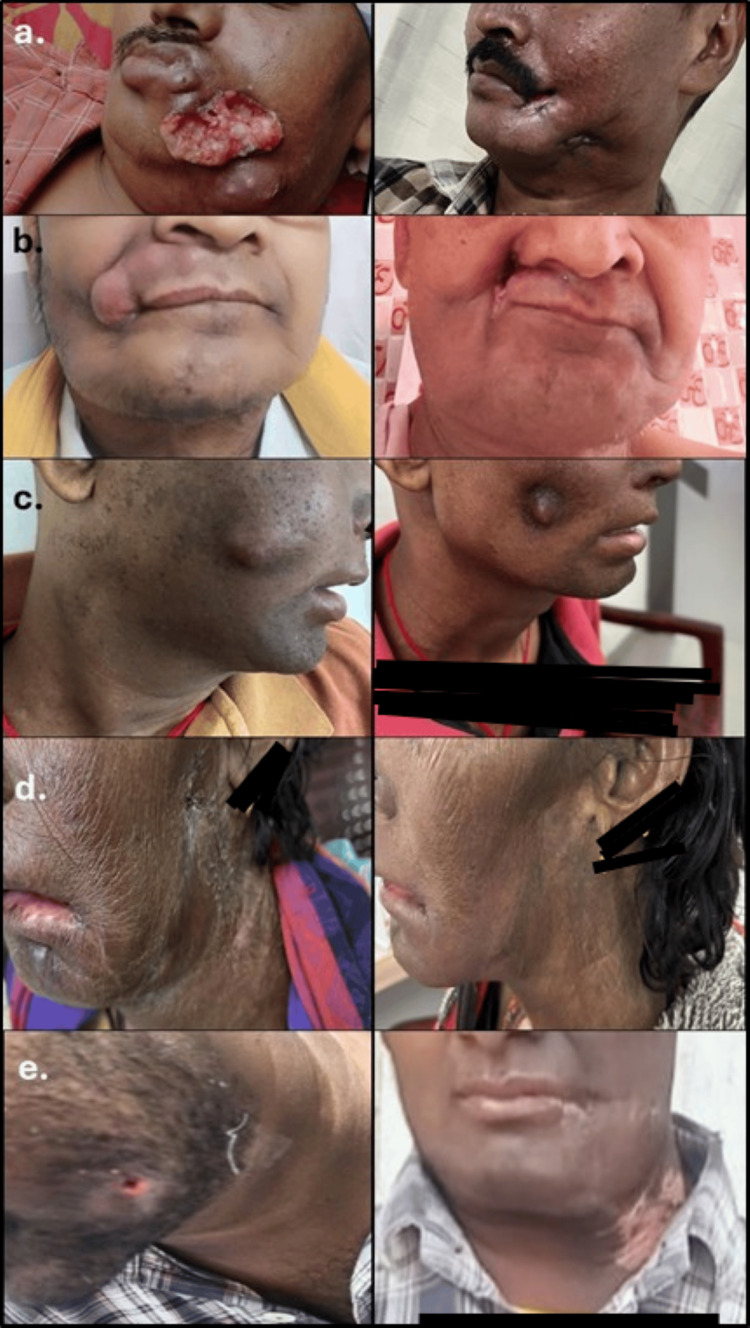
a. Case 1: Locally advanced carcinoma of the buccal mucosa, treated with palliative OMCT and biosimilar nivolumab; b. Case 2: Squamous cell carcinoma (SCC) of the hard palate, treated with palliative OMCT and biosimilar nivolumab; c. Case 3: Squamous cell carcinoma (SCC) of the buccal mucosa, treated with palliative OMCT and biosimilar nivolumab; d. Case 4: Recurrent squamous cell carcinoma of the head and neck (SCCHN), treated with palliative OMCT and biosimilar nivolumab; e. Case 5: cT4b N1 unresectable carcinoma of the buccal mucosa, treated with neoadjuvant chemotherapy (NACT, DCF regimen) and biosimilar nivolumab. OMCT: oral metronomic chemotherapy; SCC: squamous cell carcinoma; SCCHN: squamous cell carcinoma of the head and neck; cT4b N1: clinical tumour stage T4b with N1 regional lymph node involvement (TNM staging system); NACT: neoadjuvant chemotherapy; DCF: docetaxel, cisplatin, and 5-fluorouracil regimen

A 44-year-old male, a chronic smoker, presented with locally advanced carcinoma of the buccal mucosa. Tobacco smoking is an established risk factor for oral cavity SCCHN, associated with higher rates of recurrence and inferior survival [2, 14]. The patient received one cycle of cisplatin + 5-FU as initial systemic therapy but discontinued due to severe neutropenia (Grade ≥3 hematologic toxicity), a recognized complication of platinum-FU doublet therapy [9, 10]. After disease progression on alternative therapy and subsequent failure of the paclitaxel + carboplatin + cetuximab (PCC) regimen [15], the patient was deemed to have exhausted conventional options. The patient was initiated on OMCT combined with low-dose biosimilar nivolumab (40 mg, q3w). After four cycles, clinical evaluation demonstrated rapid clinical improvement, significant reduction in the ulcerative lesion, and a PR on imaging on contrast-enhanced CT (CECT) of the neck, as per Response Evaluation Criteria in Solid Tumours (RECIST) v1.1 criteria (Figure 1a) [16]. Treatment is ongoing with good tolerability and no significant immune-related adverse events [17].

Case 2: hard palate carcinoma (SCCHN) with locoregional recurrence and lung metastasis

A 56-year-old male presented with carcinoma of the hard palate. He had defaulted adjuvant therapy following initial definitive treatment and subsequently developed locoregional recurrence with lung metastasis, representing the R/M disease state associated with poor prognosis [3]. The patient's poor performance status and significant comorbidities, diabetes mellitus and hypertension, precluded the use of platinum-based systemic chemotherapy, a common real-world challenge in this patient population [9]. Given the above constraints, OMCT + low-dose biosimilar nivolumab (40 mg, q3w) was initiated as a tolerable alternative. After three cycles, the patient achieved a notable reduction in tumor burden and symptomatic improvement with planned reassessment by CECT of the neck and chest (Figure 1b). Therapy is being continued with planned radiological reassessment.

Case 3: buccal mucosa squamous cell carcinoma (SCC); early recurrence after definitive chemoradiotherapy 

A 40-year-old male with buccal mucosa SCC achieved a complete response (CR) following definitive concurrent chemoradiotherapy (CTRT) [18]. However, he developed early locoregional recurrence at six months (progression-free survival (PFS): 6 months), with the disease deemed unresectable. After progression on the PCC regimen [15], the patient's disease was refractory to multiple prior lines of treatment. The patient was treated with OMCT + biosimilar nivolumab at 3 mg/kg (standard dose) q2w [6]. After four cycles, CECT of the neck and chest demonstrated marked tumor reduction, qualifying as a PR per RECIST v1.1 (Figure 1c) [16]. The patient is continuing treatment with sustained response and good tolerability.

Case 4: Recurrent SCCHN with cachexia and poor performance status

A 52-year-old female with prior surgery and adjuvant CTRT [18] presented with recurrent disease at approximately 1.3 years after initial treatment (PFS: ~1.3 years). She exhibited significant cachexia and a deteriorated performance status, rendering her unfit for systemic cytotoxic chemotherapy [3]. OMCT + low-dose biosimilar nivolumab (40 mg, q3w) was initiated with a dual objective of disease control and performance status recovery. After four cycles, the patient demonstrated significant symptomatic improvement and meaningful recovery of performance status, enabling sustained clinical benefit (Figure 1d). Given the patient's fragility, radiological reassessment had not yet been performed at the time of this report; however, clinical response remained evident.

Case 5: CT4B N1 unresectable carcinoma of the buccal mucosa; neoadjuvant conversion to resectability 

A 42-year-old male presented with cT4b N1 unresectable carcinoma of the buccal mucosa [3]. Resectability in this context is defined by adherence to critical structures, including the masticator space and pterygoid plates, posing a surgical challenge requiring neoadjuvant tumor volume reduction [19]. The patient received neoadjuvant DCF chemotherapy combined with low-dose biosimilar nivolumab (40 mg, q3w), administered concurrently to exploit synergy between cytotoxic immunogenic cell death and PD-1 blockade [20]. The patient demonstrated a marked clinical and radiological response per RECIST v1.1, enabling conversion from unresectable to resectable disease (Figure 1e) [16]. Curative intent surgery was subsequently performed. Final histopathological staging showed ypT2 N0, confirming significant pathological downstaging. The patient is currently on adjuvant chemoradiotherapy and remains clinically stable [18].

## Discussion

This case series illustrates the potential of combining the OMCT metronomic regimen with low-dose nivolumab in a spectrum of SCCHN presentations that pose significant therapeutic challenges. To our knowledge, this represents one of the early descriptive reports of this combination in recurrent/metastatic and locally advanced oral cavity SCCHN.

Metronomic chemotherapy exerts pleiotropic anti-tumor effects beyond direct cytotoxicity, including suppression of tumor angiogenesis, inhibition of regulatory T-cells (Tregs), and direct stimulation of anti-tumor immunity, a mechanistic profile highly complementary to checkpoint blockade [20]. Methotrexate at low doses disrupts folate-dependent nucleotide synthesis and has well-established activity in SCCHN as a palliative single agent [10]. Erlotinib, an EGFR tyrosine kinase inhibitor, targets a pathway frequently dysregulated in SCCHN [11,21]. Celecoxib, a selective COX-2 inhibitor, abrogates prostaglandin E₂-mediated immune suppression within the tumor microenvironment [12].

Standard nivolumab dosing (3 mg/kg or 240 mg q2w or 480 mg q4w) in SCCHN was established based on receptor occupancy and pharmacokinetic modeling, not on linear dose-response data [7]. Emerging evidence suggests that flat doses as low as 40 mg achieve comparable PD-1 receptor saturation in many patients, with a substantially reduced cost burden, a critical consideration in resource-limited settings [8]. All patients in our series tolerated the low-dose regimen without significant immune-related adverse events, consistent with published low-dose ICI data in other tumor types [8].

In patients who are ineligible for platinum therapy (Cases 2 and 4), OMCT + nivolumab provided a meaningful treatment option with demonstrable clinical benefit, including performance status recovery. Performance status improvement on systemic therapy, as observed in Case 4, represents a clinically significant endpoint with implications for quality of life and subsequent therapeutic options [3]. In multi-refractory patients (Cases 1 and 3), the combination demonstrated activity despite prior failure of ICIs (PCC + cetuximab) [15] and platinum-based regimens [4], suggesting non-cross-resistant mechanisms with emerging dual ICI approaches. This is biologically plausible given the distinct targets of OMCT components versus prior therapies [11, 12].

The neoadjuvant conversion observed in Case 5 is particularly noteworthy. Addition of nivolumab to DCF chemotherapy may enhance immunogenic cell death, amplify T-cell priming, and augment the probability of pathological downstaging [20]. The ypT2N0 outcome represents a significant pathological response, analogous to paradigms established in other solid tumors where neoadjuvant ICI augments pathological complete response rates [19]. This is further supported by the evolving evidence base for neoadjuvant approaches in SCCHN [18, 19].

This case series carries inherent limitations of retrospective, uncontrolled case reports: small sample size, absence of a comparator arm, heterogeneity of prior therapies, and variable follow-up. Radiological assessment was unavailable in one patient (Case 4), limiting objective response evaluation. The receptor saturation data supporting 40 mg flat dosing is derived predominantly from pharmacokinetic modeling and simulation studies, rather than prospective randomized head-to-head efficacy trials. Clinical validation of this dosing strategy in SCCHN requires further investigation through adequately powered prospective clinical trials.

## Conclusions

This real-world case series demonstrates the feasibility of biosimilar nivolumab (Tishtha)-based combination strategies in advanced oral cavity and SCCHN across salvage, frail-patient, and neoadjuvant settings. Clinical responses, symptomatic benefit, and acceptable tolerability observed in these difficult-to-treat cases underscore the potential role of pragmatic immunotherapy combinations in expanding access to effective cancer care in low- and middle-income settings. Prospective evaluation in larger, systematically studied cohorts is warranted.
